# Supplementary material for: Identification of photoperiod-induced specific miRNAs in the adrenal glands of Sunite sheep (Ovis aries)
Source: Front Vet Sci. 2022 Jul 22;9:888207. doi: 10.3389/fvets.2022.888207 (PMC9354845; doi:10.3389/fvets.2022.888207)
Supplement: Supplementary file 1 [file Data_Sheet_1.ZIP › Supplementary file/miRDeep2 file.zip!/miRDeep2 file/Novel_Structure/LP42A2/Novel_65.pdf]

Provisional ID	:	NC_019484.2_37559
Score total	:	1.8
Score for star read(s)	:	-1.3
Score for read counts	:	0
Score for mfe	:	1.5
Score for randfold	:	1.6
Score for cons. seed	:	
Total read count	:	32
Mature read count	:	31
Loop read count	:	0
Star read count	:	1

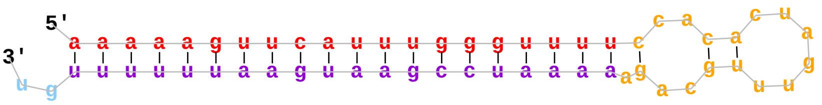

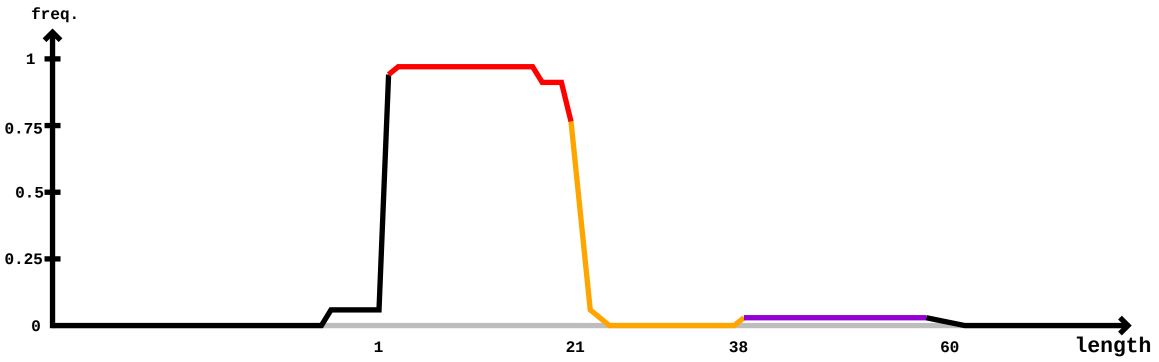


```
                                Mature                          Star
5'- gaauacuacaauaaaaaaaauauuguguuggccaaaaaguucauuuggguuuuccacacuaguuugcagaaaaauccgaaugaauuuuuuguccaacucaguaguaugca  -3'  obs
    gaauacuacaauaaaaaaaauauuguguuggccaaaaaguucauuuggguuuuccacacuaguuugcagaaaaauccgaaugaauuuuuuguccaacucaguaguaugca       exp
    ..(((((((..............((.(((((.((((((((((((((((((((((..((......))..).))))))))))))))))))))).))))).)))))))))...   reads   mm   sample
    ...........................uuggGcaaaaaguucauuuggg.............................................................   2       1    seq
    .................................aaaaaguucGuuuggguuu..........................................................   5       1    seq
    .................................aaaaaguucGuuuggguuuu.........................................................   24      1    seq
    .................................aaaaaguucauuuggguuuucU.......................................................   1       1    seq
    ..................................aaaaguucauuuggguuuuUca......................................................   1       1    seq
    ......................................................................aaaauccgaaugaauuuuuu....................   1       0    seq
```
